# The Mouse Superior Colliculus: An Emerging Model for Studying Circuit Formation and Function

**DOI:** 10.3389/fncir.2018.00010

**Published:** 2018-02-13

**Authors:** Shinya Ito, David A. Feldheim

**Affiliations:** ^1^Santa Cruz Institute for Particle Physics, University of California, Santa Cruz, Santa Cruz, CA, United States; ^2^Department of Molecular, Cell and Developmental Biology, University of California, Santa Cruz, Santa Cruz, CA, United States

**Keywords:** superior colliculus, mouse, retinotopic map, electrophysiology, sensorimotor systems

## Abstract

The superior colliculus (SC) is a midbrain area where visual, auditory and somatosensory information are integrated to initiate motor commands. The SC plays a central role in visual information processing in the mouse; it receives projections from 85% to 90% of the retinal ganglion cells (RGCs). While the mouse SC has been a long-standing model used to study retinotopic map formation, a number of technological advances in mouse molecular genetic techniques, large-scale physiological recordings and SC-dependent visual behavioral assays have made the mouse an even more ideal model to understand the relationship between circuitry and behavior.

## Introduction

The superior colliculus (SC) is an integrative sensorimotor structure that receives inputs from multiple sensory modalities and integrates them to control innate behaviors; in the mouse these include coordinating eye and head movements (Sparks et al., 1990), suspension of locomotion (Liang et al., 2015), and escape or freezing in response to a looming object (Shang et al., 2015; Wei et al., 2015). This review focuses on the recent progress in understanding the structure and function of the mouse SC that suggests that it has an expanded role in visual processing compared to its primate counterpart.

A number of features of the mouse SC suggest that it processes visual information differently than the primate SC. One major difference between the mouse and primate SC is the proportion of retinal ganglion cells (RGCs) that project to it. In mice 85%–90% of RGCs project to the SC (Ellis et al., 2016), whereas only ~10% of primate RGCs project to the SC (Perry and Cowey, 1984; Dhande and Huberman, 2014). Furthermore, the visual response properties of the RGC inputs differ between mouse and primate; for example the mouse SC receives inputs from direction selective (DS) RGCs that do not have a primate counterpart (Weng et al., 2005; Field and Chichilnisky, 2007). Conversely, the most abundant RGC type in the primate, the midget cells, which comprise approximately 80% of the primate RGCs (Perry et al., 1984), have no analog in the mouse retina (Zhang et al., 2012). The function(s) of mouse and primate SC may also differ. The primary function of the primate SC is to shift the gaze of the animal toward an interesting object so that the animal can visualize it in greater spatial detail with its fovea (Sparks, 1986). Mice use the SC to control eye/head movements, but its purpose is unclear, as they do not have a fovea. The mouse SC is involved in promoting innate defensive behaviors such as escaping or freezing (Yilmaz and Meister, 2013; Liang et al., 2015; De Franceschi et al., 2016) and this may also be true in primates (DesJardin et al., 2013). Whether the SC is involved in promoting similar defensive behaviors in other species is not known.

## Structure and Development of the Mouse SC

### The Superior Colliculus Is a Three-Dimensional Structure with Sensory Inputs Organized into a Series of Laminae, Each of Which Is Topographically Mapped and Aligned with Respect to the Visual Field

**Figure 1:**
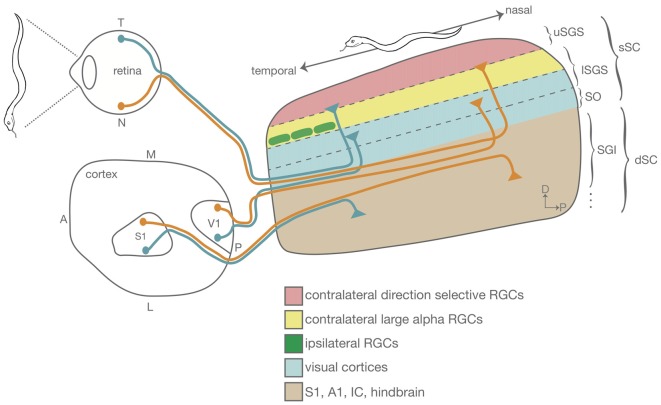
Schematic showing the organization of the mouse superior colliculus (SC) emphasizing the lamination and topographic alignment of inputs. A snake detected along the nasal–temporal axis of the retina is topographically represented along the anterior–posterior axis of the SC. Retinal ganglion cells (RGCs) send direct visual information to the superficial SC (sSC). Some RGC types segregate into sublamina within the sSC. Shown here are the direction selective (DS) RGCs that project to the most superficial lamina in the SC (uSGS, pink) and large alpha RGCs that project to a slightly deeper sSC lamina (lSGS, yellow). The SC also receives inputs from brainstem and cortical areas into the deep SC (dSC). Shown are inputs from V1 (blue) and S1 (brown), that sort such that they align cortical maps of vision and touch with the superficial retinocollicular map. This figure is not drawn to scale. A, anterior; P, posterior; T, temporal; N, nasal; S1, primary somatosensory cortex; V1, primary visual cortex; uSGS, upper stratum griseum superficial; lSGS, lower stratum griseum superficial; SO, stratum opticum; SGI, stratum griseum intermedium.

The mouse SC, as in all mammals, is organized into several synaptic layers, each of which has distinct sources of innervation (May, 2006; Basso and May, 2017; Figure 1). The most superficial lamina of the SC, the stratum griseum superficiale (SGS), receives direct RGC inputs from the contralateral retina in its most superficial region; different RGC types terminate in different sublaminae within the SGS. For example, On-Off DS RGCs project to the upper SGS (uSGS), while alpha RGCs project to the lower SGS lamina (lSGS; Huberman et al., 2008b, 2009; Kim et al., 2010; Dhande and Huberman, 2014; Martersteck et al., 2017). The lSGS also receives visual input from the ipsilateral retina and the primary and extrastriate visual cortex (Dräger and Olsen, 1980; Wang and Burkhalter, 2013). The deeper SC (stratum griseum intermedium, SGI and below; dSC) is also laminated and receives inputs from the superficial SC (stratum opticum, SO and above; sSC; Gale and Murphy, 2014), primary motor, somatosensory (S1) and auditory cortex (A1; Dräger and Hubel, 1975b; Triplett et al., 2009, 2012; Zingg et al., 2017), as well as brainstem nuclei such as the brachium of the inferior colliculus (Wallace and Fredens, 1989).

Information about how the SC forms its stereotypical laminated structure during mouse development is limited, but it is hypothesized to use a combination of molecular and activity dependent cues. Time-course studies of the ingrowth of labeled On-Off DS RGCs (labeled by the TRHR-GFP and Drd4-GFP lines), V1 and S1 axons show that axons grow into the SC in a lamina-restricted manner rather than sampling all laminae before making a decision (Triplett et al., 2009, 2012; Osterhout et al., 2014). Results from investigators studying the development of the zebrafish tectum (analogous to the vertebrate SC) demonstrated that laminar specificity develops via a combination of axon guidance cues and cell adhesion mechanisms (Xiao et al., 2011; Antinucci et al., 2013). It seems likely that similar mechanisms are used to direct incoming axons to their stereotypical laminae in the mouse SC. Several axon guidance and cell adhesion molecules are expressed in a lamina-restricted manner in the mouse SC (Byun et al., 2016), but whether and how they act to guide incoming axons to their proper targets remains unknown.

Axon–axon competition and neural activity may also be used to refine axonal inputs into laminae. Although lamination of Off-alpha RGCs labeled by the CB2-GFP mouse line is unchanged in the β2-nicotinic acetylcholine receptor mutant mouse (a mouse that has altered spontaneous activity patterns in the retina during development, see “A Combination of EphA/ephrin-A Signaling and Patterned Spontaneous Retinal Activity Is Used to Form a Retinocollicular Map” section), V1 axons project to a deeper SC lamina and are more segregated from the retinal inputs in these mice. In addition, removal of contralateral RGC input to the SC results in ipsilateral RGC axons and V1–SC axons to project to a more superficial location within the SGS (Land and Lund, 1979; Triplett et al., 2009; Maiorano and Hindges, 2013). This may mean that correlated neural activity provided by the retina is used to overcome a laminar barrier or is needed to read a laminar-derived cue. One intriguing hypothesis is that neural activity can modulate cadherin expression; down regulation of adhesion is necessary for branching into adjacent laminae. Consistent with this idea, a gene expression profiling study found that cadherin 1 (Cdh1) expression is suppressed in the lateral geniculate nucleus (LGN, a thalamic target of RGCs) when retinal activity is perturbed (Rubin et al., 2011).

### Each Lamina in the SC Is Arranged to Represent a Topographic Map of Visual Space

Within each SC lamina, inputs are mapped topographically with respect to the visual field, and are aligned in retinotopic register with each other. Contralateral RGCs that project to the most superficial lamina of the SC are organized such that the temporal–nasal (T–N) axis of the retina projects along the anterior–posterior (A–P) axis of the SC, and the dorsal–ventral (D–V) axis of the retina projects along the lateral–medial (L–M) axis of the SC. Ipsilateral RGCs and V1 axons terminate in a slightly deeper SGS lamina and are retinotopically aligned with the contralateral retinocollicular map. SC inputs from the primary somatosensory cortex (S1), auditory brainstem and cortex, and other brainstem nuclei such as the inferior colliculus and trigeminal nucleus project to a deeper SC location than V1 axons in the SGI. Each of these projections is organized such that neurons from different sensory modalities that monitor the same portion of the environment terminate in register but in distinct laminae (Figure 1).

### A Combination of EphA/ephrin-A Signaling and Patterned Spontaneous Retinal Activity Is Used to Form a Retinocollicular Map

Experiments in the last decade have revealed the mechanisms by which RGC axons sort to form a topographic map in the SC, especially along the nasal–temporal (azimuthal) axis of the visual field (reviewed by: Triplett and Feldheim, 2012; Seabrook et al., 2017). The generation of topography along this axis requires a combination of Eph/ephrin signaling and patterned spontaneous retinal activity (Huberman et al., 2008a; Feldheim and O’Leary, 2010). EphA receptor tyrosine kinases and their ligands, ephrin-As, are expressed in counter gradients along the T–N axis of the retina and the A–P axis throughout the depth of the SC (Cang et al., 2005; Rashid et al., 2005; Triplett et al., 2012). EphA/ephrin-A interactions are largely repulsive (but may also be attractive, see Hansen et al., 2004) and result in temporal RGCs terminating in the anterior SC, and nasal RGCs terminating in the posterior SC (Drescher et al., 1995; Nakamoto et al., 1996; Monschau et al., 1997; Rashid et al., 2005). EphA/ephrin-A signaling is essential for normal map development. Disruption of EphA/ephrin-A signaling results in RGC axons having ectopic termination zones and SC neurons having topographically incorrect receptive field (RF) locations that are limited to the A–P axis in the SC (Frisén et al., 1998; Brown et al., 2000; Feldheim et al., 2000; Pfeiffenberger et al., 2006; Cang et al., 2008b; Triplett et al., 2009). RGC type-specific lamination in the SC is preserved in ephrin-A mutant mice, demonstrating that topography and lamination develop via independent mechanisms (Sweeney et al., 2015).

Correlated neuronal activity is also required for retinocollicular map formation (McLaughlin et al., 2003). During retinal development, waves of activity propagate across the retina and drive corresponding waves in the visual cortex and the SC (Meister et al., 1991; Ackman et al., 2012). During the first postnatal week in mice, these waves require acetylcholine for their propagation; in the absence of the β2 subunit of the nicotinic acetylcholine receptor, waves are disrupted, resulting in anatomical and functional defects in topography. For example the RGC axons of β2 mutants terminate in their approximately correct topographic position, but do not refine into a discrete termination zone. Consistent with this loss of anatomical refinement, the SC neurons in β2 mutant mice have larger RFs (McLaughlin et al., 2003; Chandrasekaran et al., 2005). Interestingly, these perturbations are observed specifically in the azimuthal axis; this is consistent with the findings that showed that retinal waves predominantly propagate along the nasal-temporal retinal axis (Stafford et al., 2009; Ackman et al., 2012). Mice that lack ephrin-As or those that have altered retinal wave patterns each maintain some retinocollicular topography; however, a combination of these ephrin-A/β2 mutations leads to dramatic defects in both anatomical and functional topography (Pfeiffenberger et al., 2006).

One conclusion from the findings that errors in topography in ephrin-A mutants are restricted to the N–T mapping axis is that the generation of D–V topography uses ephrin-A independent mechanisms to form (Cang et al., 2008a). Although the details of D–V mapping mechanisms are not as well understood as those for N–T mapping, current evidence suggests that a combination of pre-target sorting and EphB/ephrin-B signaling is used to establish topography along the M–L SC axis (Triplett and Feldheim, 2012; Seabrook et al., 2017). As RGC axons approach the SC they defasciculate and sort such that D–V order is already established prior to axons entering the SC (Simon and O’Leary, 1991; Plas et al., 2008). This order is diminished in mice that have altered BMP signaling in the developing retina and is concomitant with defects in D–V topography in the SC (Plas et al., 2008).

Evidence supporting a requirement for ephrin-B/EphB signaling in D–V mapping comes from *in vivo* studies in which EphBs have been removed. In EphB2/B3 double knockout mice, DiI tracing of ventral RGC axons showed that in addition to a largely correct termination, ectopic termination zones are also formed (Hindges et al., 2002). This mapping defect is not as dramatic as those seen in EphA/ephrin-A mutant mice, suggesting that other yet undiscovered mechanisms exist to help map the D–V axis.

### Mechanisms of SC Map Alignment

A developmental challenge of SC is to ensure that inputs coming in from distinct sources terminate such that axons that originate from different sensory areas but refer to the same location in space are aligned (Anishchenko and Feller, 2009). As with topographic mapping within a lamina, incoming axons are aligned by a combination of graded molecular cues and activity-dependent mechanisms.

Evidence suggests that the contralateral RGC map instructs ipsilateral RGC and layer 5 V1 axons where to synapse in the lSGS to ensure that their visual RFs will overlap. When contralateral RGCs are removed early in development via enucleation or using an Atoh7 (Math5) mutant mouse (these mice fail to develop RGCs; Brown et al., 2001), both ipsilateral (Reese, 1986) and V1 (Triplett et al., 2009) projecting axons fail to refine to their topographically correct location. Consistent with this result, genetic manipulations that altered the topography of the contralateral RGC map (via ectopic expression of EphA3 in a subset of RGCs, EphA3 knock-in (EphA3ki) mouse; Brown et al., 2000) result in the rearrangement of V1 axonal projections in order to maintain alignment with the RGC map (Triplett et al., 2009). This rearrangement does not occur in β2 mutant mice, leading to a model whereby V1 axons terminate in the SC by matching activity patterns derived from retinal waves that propagate throughout the visual system during development (Ackman et al., 2012; Ackman and Crair, 2014). A different experiment suggests that EphA/ephrin-A interactions between incoming V1 axons and RGC axons in the SC are also used to align these maps. When ephrin-A3 is ectopically expressed in a subset of RGC axons there is no defect in retinocollicular topography, but the V1–SC map is disrupted in a manner consistent with axonal ephrin-A3 acting as a repellent for incoming V1 axons (Savier et al., 2017).

The dSC receives inputs from the ears and body; these also map topographically, resulting in neurons in the dSC that respond to sound, touch and/or light when presented in the same part of space (Dräger and Hubel, 1975a,b, 1976). Classic experiments in the barn owl tectum showed that retinal input is instructive for precise auditory/visual alignment (Knudsen and Knudsen, 1989a,b). When barn owls were fitted with prismatic goggles that optically displace the visual field onto the retina, there is a misalignment between the visual and auditory maps in the tectum. During a sensitive period in early life, these prism-reared owls are able to realign their auditory map to match the visually displaced retinal map. While much less is known about how dSC neurons align with the visual map in the mouse, it is known that a retinal template matching mechanism does not explain S1–SC mapping. Unlike V1 axons, S1 axons do not rearrange their projections to match the altered retinal map of the EphA3^ki^ mouse, and enucleation does not affect the S1 axon termination pattern (Triplett et al., 2012).

## Visual Response Properties of the Mouse SC Neurons

### Neurons in the Mouse SC Are Selective to Visual Features

Although the architecture of the mouse SC is similar to that of primates, the visual response properties of mouse and primate SC neurons are different. In the primate, SC neurons respond to visual stimuli within their RF regardless of the specific features of the stimulus. This type of neuron is often called an event detector. Event detector cells are the most numerous in the superficial primate SC and are not selective to specific directional movement, orientation, or shape of the stimulus (Humphrey, 1968; Schiller and Koerner, 1971; Cynader and Berman, 1972; Goldberg and Wurtz, 1972). Their transient responses are suited for encoding the location of a novel object that is visually salient. On the other hand, mouse SC neurons act more like “feature detectors” in that a specific subset of SC neurons responds best when a specific type of stimulus is presented within its RF. These neurons might be useful for detecting visual features of a potential threat and immediately respond by initiating a defensive behavior without further analysis of the visual scene. A number of recent studies have characterized the visual response properties of SC neurons using various visual stimuli (Wang et al., 2010; Gale and Murphy, 2014, 2016; Zhao et al., 2014; Inayat et al., 2015; Ito et al., 2017). Unlike the primate SC neurons, the mouse SC neurons exhibit a number of different response properties. The types of visual stimuli used for these studies are summarized in Table 1. Below is a summary of the responses elicited by each stimulus.

**Table 1:** List of stimuli used for measuring visual response properties of mouse superior colliculus (SC) neurons.

Visual stimulus	Identified response types and properties	Other remarks	References
Flashing spot	On, Off, On-Off response types, On/Off RF overlap, RF sizes	Most cells are On-Off with overlapping RFs. Deeper neurons have large RFs.	Wang et al. (2010), Gale and Murphy (2014), Inayat et al. (2015) and Ito et al. (2017)
Moving spot	Selectivity to small moving spot	WF cells strongly respond to small moving spots	Gale and Murphy (2014)
Drifting gratings	OS/DS (positive/negative), complex-cell-like (C-like) nonlinearity	Most cells are C-like nonlinear. DS is enriched in the very superficial SC	Wang et al. (2010), Gale and Murphy (2014), Inayat et al. (2015) and Ito et al. (2017)
Looming spot	Looming spot responsiveness, tuning to looming speed	Cortex modulates response gain, but not speed tuning	Zhao et al. (2014)
Contrast modulated noise movie	Stimulated-by-contrast cells, suppressed-by-contrast cells	Suppressed-by-contrast is minority, but increase in the deep area	Ito et al. (2017)
Contrast reversing gratings	Y-like nonlinearity	Exclusively in sSC	Ito et al. (2017)

#### Flashing (Stationary) Spot

A flashing light or dark spot on a gray background (Ito et al., 2017) or a flashing light spot on a dark background (Wang et al., 2010). This stimulus is used for determining the location and size of the RF as well as whether the neuron responds to the onset (On cells), or offset of a luminance change (Off cells), or to both (On-Off cells). Most of the sSC cells are On-Off cells with overlapping On and Off RFs that have a wide range of sizes (Wang et al., 2010).

#### Moving Spot

A moving light spot on a dark background. This stimulus was used to determine the response properties of the wide-field cells (defined by their morphological properties; see “Cell Classification by the Response Properties May Not be Feasible with Current Technologies” section) that selectively respond to a small spot that moves slowly. The surround suppression property can also be measured by showing two moving spots simultaneously.

#### Drifting Gratings

Sinusoidal gratings that drift across the stimulus screen. This stimulus is used to identify orientation selective (OS) or DS neurons. The same stimulus was used to identify cortical complex-cell-like spatial summation nonlinearity (C-like nonlinearity) by using the ratio between the first and zeroth temporal harmonics to the stimulus (Wang et al., 2010; Ito et al., 2017). Through a model-based analysis, Ito et al. (2017) found OS/DS neurons that respond with a negative firing rate change (negative OS/DS cells).

#### Looming Spot

A dark spot on a gray background whose diameter becomes larger over time. When presented overhead to a mouse, this stimulus induces defensive behavior (see “Behaviors Associated with the Mouse SC” section). Neurons in the sSC show robust responses to this stimulus, and their gain is modulated by cortex in awake mice (Zhao et al., 2014).

#### Contrast Modulated Noise Movie

Sinusoidal contrast modulation with 10 s period is applied to a noise movie (Niell and Stryker, 2010). This stimulus is useful for identifying the neurons that respond to high-contrast periods and low-contrast periods. The cells that fire in high-contrast periods are called stimulated-by-contrast cells; the cells that fire in low-contrast periods are called suppressed-by-contrast cells. Both stimulated-by-contrast cells and suppressed-by-contrast cells were identified in the SC (Ito et al., 2017).

#### Contrast Reversing Gratings

Sinusoidal gratings that change their contrast periodically over time. This stimulus has been used to identify retinal Y-cells that have nonlinear spatial summation (Hochstein and Shapley, 1976). The Y-like nonlinear cells were found in the SC, exclusively in the sSC (Ito et al., 2017).

The purpose of having a large variety of visual responses in the mouse SC neurons is unclear, but this likely gives the SC the ability to analyze visual scenes instead of simply identifying a salient object. Note that one neuron can have more than one of the response properties listed above. For example, a DS cell can also have Y-like spatial nonlinearity. It remains unknown if having one property affects the probability of having another property.

### The Visual Response Properties of SC Neurons Differ between Laminae

Because RGCs terminate in different SC laminae, it is expected that SC response properties also differ between laminae. Within the sSC, Inayat et al. (2015) found that DS cells are enriched in the very superficial part of the sSC, where DS-RGC axons project. Ito et al. (2017) made a quantitative comparison of the visual responses between the sSC and dSC and identified a number of differences in the response properties of these two areas. The sSC is enriched with cells that have small RFs, high evoked firing rates, and sustained temporal responses with early onsets. In contrast, the dSC is enriched with the negative OS/DS cells and cells with large RFs, low evoked firing rates, and transient temporal responses with late onsets. Almost all of the dSC cells have C-like nonlinearity, but the cells with Y-like nonlinearity are present only in the sSC.

### Feature Selectivity Is Generated by Both Retinal and Non-retinal Inputs to the SC

Determining the inputs that generate the feature selectivity of SC neurons is key to understanding SC function. In theory, feature selectivity could be inherited directly from the retina, derived within the SC, or derived from descending inputs from other brain areas. Studies suggest that each of these mechanisms contributes to the feature selectivity of the SC neurons.

The mouse retina has ~32 distinct RGC types that have been identified physiologically using clustering methods of the response properties of more than 11,000 RGCs recorded using calcium imaging; however, the contribution of each type to the response properties of SC neurons is not known (Baden et al., 2016). Recently, it has been shown that DS SC neurons get their properties directly from On-Off DS RGCs. On-Off DS RGCs are generated in the retina via asymmetric inhibition by starburst amacrine cells (Wei et al., 2011). Shi et al. (2017) showed that genetically blocking inhibition from starburst amacrine cells leads to a decrease in both DS RGCs and DS SC neurons, suggesting that the retina is the origin of DS response properties in the SC. As for the OS response, at least two distinct types of OS RGCs have been described in the mouse retina (Nath and Schwartz, 2016) and therefore SC OS cells could get their properties from these RGCs. Future experiments are needed to test this hypothesis.

Some visual features are generated within the SC. Gale and Murphy (2016) showed that inhibitory inputs from horizontal SC cells onto wide-field cells are used to shape the wide-field cell’s RF such that it best responds to a small moving object. When the horizontal cell’s activity was suppressed via optogenetics, the wide-field cell’s response selectivity was diminished and it started to respond to larger or stationary stimuli.

While the cortex does not seem to create feature selectivity in SC responses, it does modulate them. Removing or silencing the visual cortex does not affect the formation of On-Off, DS, or OS responses (Zhao et al., 2014; Shanks et al., 2016). However, optogenetic inhibition of V1 neurons does change the gain of the SC neuron’s response to a looming spot in awake (but not in anesthetized) mice (Zhao et al., 2014).

The behavioral state of an animal can also modulate the visual responses of the SC neurons. Ito et al. (2017) found that more than half of the visually responsive neurons receive an additive (a constant shift of the firing rate) and/or multiplicative (change in the firing rate gain to the stimulus) modulation in response to drifting grating stimuli while a mouse is moving compared to the mouse at rest. Other neurons receive more complex modulation that includes a shift, mainly downward, in their preferred spatial frequency of the drifting gratings. These modulations are different from those reported in mouse V1 where the cells do not change their preferred spatial frequencies, and the cells that prefer a high spatial frequency receive a higher gain modulation (Mineault et al., 2016). The origin of this locomotion-related modulation to the SC is not known.

### Cell Classification by the Response Properties May Not be Feasible with Current Technologies

Despite the success of the classification of RGCs with large-scale recordings (~11,000 neurons) with calcium imaging (Baden et al., 2016), a similar approach to classifying SC cell types seems challenging. Because 85%–90% of the RGCs project to the SC, we can assume that a large fraction of the 32 RGC types also project to the SC. As each SC cell receives innervation from an average of 5.5 RGCs (Chandrasekaran et al., 2007), unless a given SC cell receives projections only from the same or a few RGC types, the potential number of distinct visual responses observed in the SC could be quite large. In addition, the internal circuitry in the SC (studied *in vitro* in: Phongphanphanee et al., 2008, 2011; Isa and Hall, 2009) and connections from other brain areas (V1: Dräger and Hubel, 1976; parabigeminal nucleus: Mufson et al., 1986; substantia nigra: Kaneda et al., 2008) could create yet further complexity of the visual response patterns of SC neurons. Therefore, unless there is specificity in RGC inputs into the mouse SC neurons, a comprehensive classification using current recording technology may not be feasible.

**Figure 2:**
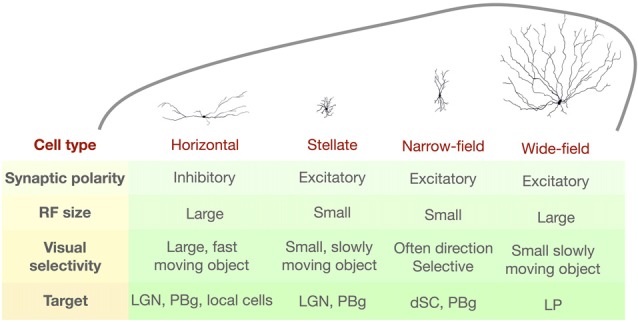
Summary of the morphological and functional properties of four distinct SC cell types as defined by Gale and Murphy (2014). RF, receptive field; LGN, lateral geniculate nucleus; PBg, parabigeminal nucleus; dSC, deep SC; LP, lateral posterior nucleus.

An alternative approach is to classify the SC neurons using morphological and/or intrinsic membrane property criteria. An early attempt to do this in the rat SC identified five morphologically distinct SC cells types (Langer and Lund, 1974). In the mouse, Gale and Murphy (2014) identified four distinct cell types in the superficial SC: wide-field cells, horizontal cells, stellate cells and narrow-field cells. This classification scheme is useful because these cell types also differ in the target structures to which they project, suggesting that the features they detect are important for different behaviors (see “Behaviors Associated with the Mouse SC” section). The visual stimulus preferences and the projection targets for each type are summarized in Figure 2.

### Orientation Selectivity Is Heterogeneously Distributed in the Mouse SC

It has recently been found that the OS neurons in the mouse SC are not distributed homogeneously across the transverse extent of the SC, meaning that the SC is regionalized with respect to the features it detects. Feinberg and Meister (2015) used two-photon calcium imaging to measure the activity of posterior SC neurons in response to moving bars. They found that large patches (>200 μm diameter) of SC neurons share a similar orientation preference, and this preference is preserved throughout a column of cells in the uSGS. Another set of experiments used both calcium imaging and extracellular recordings to show that OS cells prefer concentric orientations around the center of the visual field such that topographically distinct locations respond to different bar orientations (Ahmadlou and Heimel, 2015). While these studies differ somewhat in their conclusions about SC structure, a common conclusion is that different parts of the visual field are processed differently in the mouse SC. This non-uniform feature extraction may be inherited directly from the retina, as some RGC types have non-uniform distribution across the retina (Bleckert et al., 2014). Mice make saccade-like eye movements in an SC-dependent manner. It could be that mice use eye movements to put an object of interest into different parts of the visual field for feature extraction, analogous to the function of eye movements in foveated animals.

## Auditory and Somatosensory Response Properties and Multisensory Integration

Although the mouse SC receives inputs from auditory and somatosensory systems, very little is known about the response properties of the SC neurons that receive these inputs. While studied in other mammals (Chalupa and Rhoades, 1977; King and Palmer, 1983; Carlile and King, 1994; King and Carlile, 1994; Wallace et al., 1996; Gaese and Johnen, 2000) there is only a single study of the auditory, somatosensory and bi- and tri-modal neurons in the mouse dSC (Dräger and Hubel, 1975a,b). Moreover, in this case, only a limited number of cells were studied (18 cells and 27 multiunit recordings) and the best stimuli were “more complex sounds rich in high frequencies, such as clicks made by two finger nails or the crackling of cellophane”. Therefore, it is clear that future work, taking advantage of modern recording technology and genetic tools, will be aimed at understanding the properties of auditory and multimodal neurons and their development.

The cat is the best-studied animal model regarding multi-sensory integration and its development in the SC, supported by ample experimental data (Meredith and Stein, 1983, 1996; Stein and Stanford, 2008; Stein et al., 2014) and multiple mathematical models (Magosso et al., 2008; Ursino et al., 2009; Cuppini et al., 2010). These models will need to be modified for the mouse SC because of the presence of visual feature selectivity described in “Visual Response Properties of the Mouse SC Neurons” section. In addition, the auditory response properties of the mouse SC have not been characterized, and if there are differences between the auditory response properties of the mouse SC and the cat SC, they will be incorporated into these models.

Deficits in multisensory integration are known symptoms of patients with autism spectrum disorder (ASD; Iarocci and McDonald, 2006; Marco et al., 2011). Some people with ASD have deficits in temporal integration of multisensory information (Kwakye et al., 2011; Stevenson et al., 2014). Importantly, serotonin transporter (SERT) Ala56 knock-in mice, that are analogous to mutations found in some humans with ASD, have elevated serotonin levels, and are deficient in a multisensory integration task (Siemann et al., 2017). The SC is likely to have an important role in this behavioral deficiency because it is a major hub of multisensory processing, expresses multiple serotonin receptor subtypes, and receives projections from the dorsal raphe nucleus, which is a major source of serotonin (Siemann et al., 2017). Combined with the genetic and recording tools, analysis of SC response properties in mouse ASD models could lead to a better understanding of the underlying circuitry of multisensory integration and perhaps human ASD.

## Behaviors Associated with the Mouse SC

### The SC Mediates Visually-Induced Defensive Behaviors

**Figure 3:**
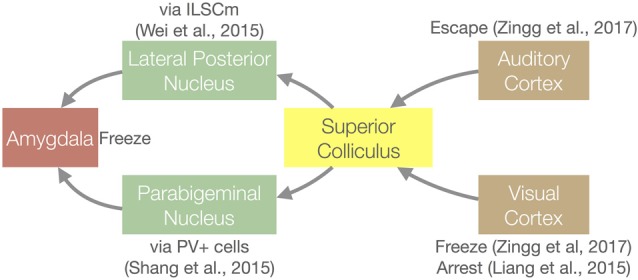
Circuit diagram for the innate defensive behaviors that involve the SC and the key articles that demonstrated each connection. See “The SC Mediates Visually-induced Defensive Behaviors” section for details. PV+, Parvalbumin expressing cells; ILSCm, medial region of the intermediate layers of the SC.

The development of molecular circuit tracing and optogenetic tools for the mouse has led to remarkable progress in understanding the function of the mouse SC (summarized in Figure 3). In mice, an overhead looming or moving spot induces innate defensive behaviors (Yilmaz and Meister, 2013; De Franceschi et al., 2016), and a flashing light can arrest locomotion (Liang et al., 2015). In order to determine the pathway leading to the looming spot response, Wei et al. (2015) used optogenetic stimulation of different brain regions to see which can induce freezing behavior. They identified a pathway that starts with the medial region of the intermediate layers of the SC and travels through the lateral posterior nucleus (mouse analog of the primate pulvinar nucleus) before being transferred to the amygdala. Another group found that parvalbumin-positive (PV+) excitatory neurons in the SC are important for the freezing response. These PV+ SC neurons project to the parabigeminal nucleus, which then project to the amygdala (Shang et al., 2015). Two other experiments show that corticocollicular inputs can induce defensive behaviors. Liang et al. (2015) demonstrated that V1–SC projections are sufficient to evoke light-induced locomotion arrest behavior. Optogenetic silencing of layer 5 V1 neurons significantly reduced light induced arrest; stimulation of SC-projecting V1 neurons induced this behavior. Zingg et al. (2017) showed that optogenetically stimulating the SC cells that receive projections from auditory and visual cortex (identified by trans-synaptic anterograde tracing from these areas) drives escape and freezing behavior, respectively. Taken together, these studies show that different types of stimuli and/or environmental context specify different behavioral outcomes, and that the SC plays a central role in differentiating these pathways using information arising from multiple sources.

### Mice Are Predators

Mice are both prey and predators. Hoy et al. (2016) demonstrated that laboratory mice hunt, capture, and eat crickets, and that both audition and vision are used for accurate cricket approach and capture. Although there is no direct evidence that the SC is involved in this behavior, the SC is likely to play a role in the cricket capture task; cricket capture requires continuous orienting behavior toward the prey, which involves inputs from both the visual and auditory pathways. Circuit tracing and stimulation studies, as done for the defensive behaviors, will determine how much and what aspects of this behavior depend on the SC or the cortex.

## Conclusion and Future Directions

Due to its importance for vision and the development of tools that record, trace, and stimulate neurons *in vivo*, the mouse SC is an emerging model for studying how circuits form, integrate, and function to produce behavior. During development, a combination of molecular and activity dependent cues sort incoming axons into their proper lamina with topographic order. SC neurons are feature detectors that are a product of the RGC inputs they receive and intracollicular projections, and their response properties are modulated by descending cortical inputs. New tools that trace and stimulate specific neuronal populations show that the SC is essential for defensive behavioral responses to visual and even auditory stimuli. Despite this progress, there is still much to learn. Molecular details about how lamination in the SC is achieved are lacking, as is an understanding of the mechanisms used to align somatosensory and auditory maps with the visual map in the SC. Also missing is information about how auditory and somatosensory information is processed and integrated in the SC. Finally, it will be important to determine if the mouse SC has unique properties compared to animals with a larger cortex and if the mouse SC is a good model for understanding deficits in multisensory integration associated with human ASD.

## Author Contributions

SI and DAF each wrote and edited this manuscript.

## Conflict of Interest Statement

The authors declare that the research was conducted in the absence of any commercial or financial relationships that could be construed as a potential conflict of interest.
